# The legal imperative for treating rare disorders

**DOI:** 10.1186/1750-1172-8-135

**Published:** 2013-09-06

**Authors:** Hanna I Hyry, Jonathan CP Roos, Jeremy Manuel, Timothy M Cox

**Affiliations:** 1 Department of Medicine, University of Cambridge, Cambridge CB2 0QQ, UK; 2 European Gaucher Alliance, UK Gauchers Association, 340 West End Lane, London NW6 1LN, UK

**Keywords:** Law, Orphan, Orphan drug, Rare disease, Human rights, Disability, Tort, Judicial review

## Abstract

**Background:**

Life-saving orphan drugs are some of the most expensive medicines. European Union governments aim to accommodate their provision within stretched healthcare budgets but face pressure to reduce funding of such treatments. Patients struggle to retain or gain access to them as their special status is questioned, causing distress and in some cases, fears of premature death. In the UK and EU reimbursement and pricing model of drugs, and orphan drugs in particular, is being re-evaluated.

**Methods:**

Using the United Kingdom as a case study we present, for the first time, legal arguments which compel governments to provide orphan medicinal products. These include (i) disability legislation, (ii) national and organisational constitutions, (iii) judicial review, (iv) tort law and (v) human rights legislation. We then address directly potential objections to our analysis and counter arguments which aim to limit provision of orphan drugs to the intended patient recipients.

**Results:**

We demonstrate that a compelling case can be made that the law demands the treatment of orphan diseases.

**Conclusions:**

Our legal framework will assist doctors and patients in ensuring the continued provision of treatments despite significant economic pressure to reduce funding. These legal avenues will empower stakeholders in drafting funding guidelines throughout the EU. The legal right to treatment extends beyond rare diseases and our analysis may therefore affect allocation of healthcare budgets throughout the EU.

## Orphan drugs at a time of austerity

Many rare, ‘orphan’, drugs are among the most expensive medical treatments [1]. High pricing results from the marketing monopolies provided by orphan drug acts to incentivise companies to develop treatments for rare diseases. However, the legislation has no price caps and funding remains the duty and prerogative of Member States [2], thus leaving individual countries to allocate health care budgets [3]. Their high cost has led to such drug provision being challenged. OECD countries’ spending on pharmaceutical products is growing faster than their total health spend [3]. In Sweden, a national health authority recently withdrew the exemption for Gaucher treatments from cost effectiveness assessment due to their “very high price” [4]. The Netherlands has questioned whether treatment for Fabry and Pompe diseases, costing up to €200,000 and €700,000 per patient per year respectively, should be fully reimbursed [5]. In view of the “continuing and mounting pressure” on the £11 billion NHS budget [6-8], the UK has adopted the Health and Social Care Act 2012 with broad provisions for reform.

## The cost of not funding orphan drugs

High prices and constrained budgets often leave patients to argue for access to what they see as life-saving treatment [9]. The burden placed on patients and their families is exacerbated further by the need to confront health care providers and engage with the impersonal world of health bureaucracy to plead for access to treatment. In Greece, fears have been expressed that stopping treatment will cause some patients to die prematurely [10].

Beyond the human toll, orphan diseases also have economic consequences. Individually rare, orphan conditions affect one in 2,000 EU citizens, equating to between 27 and 36 million patients in the EU [11]. Orphan diseases thus present a major challenge to population health and, untreated, are likely to contribute to large social and economic losses affecting more than the individual, since often family members must forego employment outside the home in order to care for their sick relatives. In contrast, treated patients, for example two individuals with Gaucher’s disease, pay income tax on successful careers in national politics and corporate law - they are unlikely to require other high cost procedures, such as pain management and surgery.

## Legal arguments for securing funding

The European Union is committed to equality of care, stressing the “paramount” need for “equity and solidarity” of care between orphan and common diseases [12], as well as requiring “delivery of high-quality, accessible and cost-effective healthcare” for those suffering from rare diseases [13].

We have previously argued that present pricing of some orphan drugs may warrant a competition law investigation [14] and that this could reduce cost, and with appropriate dialogue, preserve the necessary commercial incentives for therapeutic innovation.

Here we introduce five legal routes which physicians and patients may use to advocate for funding of treatment, and ensure that once started, it is not withdrawn: (1) disability legislation, (2) national and health system constitutions, (3) judicial review, (4) tort law and (5) human rights legislation. We examine the position in the United Kingdom as an example, and consider European Union law where relevant.

Scrutiny of this legal framework is timely. Under the UK Health and Social Care Act, the “very high cost” orphan treatments will be assessed separately from April 2013 by the National Institute of Clinical Excellence. Its aim is to devise a “robust, independent and transparent” mechanism for the commissioning of orphan products [15]. A separate Commissioning Board will have responsibility for determining the number of centres and levels of funding for patients affected by rare conditions, and ministers will retain the power to decide which services should be commissioned [15]. These mechanisms and decisions must be arrived at within the constraints of the law which we explore below. These constraints must also apply to commissioning decisions of non-orphan therapies.

### Disability legislation

Disability Legislation is enshrined by the UN Convention of 2007, EU disability legislation and in the UK, the Equality Act.

#### (i) UN Convention on the Rights of Persons with Disabilities

The 2007 UN Convention on the Rights of Persons with Disabilities [16] promotes the rights of those with disabilities including those who require intensive support [17]. Government departments of signatory countries are required to “consider what the Convention says when developing a policy or programme that affects disabled people” and to involve them when developing policies which affect them [18]. Thus a consultation period is not only advisable but mandatory; any policy implemented without regard could be challenged in the Courts.

Article 25 of the Convention ensures the right to enjoy “the *highest attainable standard of health* without discrimination on the basis of disability”, and signatories must take “all appropriate measures to ensure access for persons with disabilities to health services,” including “the *same range*, *quality and standard of free or affordable health care and programmes* as provided to other persons” (emphases added) [19].

The Convention specifically addresses the need to provide “*those health services needed by persons with disabilities specifically because of their disabilities*, *including early identification and intervention as appropriate*, *and services designed to minimize and prevent further disabilities*, *including among children*…” (emphasis added) who suffer disproportionately from orphan diseases [19]. Appropriately, the EU has designated rare diseases a priority area [12] for delivery of high-quality, accessible and cost-effective health care [13].

Additional file 1: Table S1 summarises other key provisions of the Convention, including why rare diseases likely qualify as a disability. Breach of Convention rights by a country can be appealed to the UN by virtue of the Optional Protocol to the Convention [18,20].

#### (ii) EU disability legislation

All EU countries are signatories to the UN Convention but the Union has also proposed its own further protections against discrimination for the disabled, ensuring effective access for disabled persons or groups to health care, provided that this would not provide a disproportionate burden [21]. Draft Article 11 also postulates a dialogue with relevant stakeholders of the kind envisaged by the UN Convention.

#### (iii) United Kingdom Equality Act

The UK Equality Act 2010 creates a duty on public bodies (such as NICE, the NHS and commissioning groups) to consider how their policies and service delivery will affect people with disability [22,23]. This is defined as a physical or mental impairment which substantially affects a person’s ability to perform normal day-to-day activities in the long term [24,25]. Though not mentioned explicitly, this would include rare diseases, as other progressive conditions such as multiple sclerosis, cancer and motor neurone disease are treated as a disability from the point of diagnosis [24].

Under the Act public bodies must eliminate unlawful discrimination and to advance equality of opportunity. The UK Department of Health acknowledges that this extends to healthcare commissioning decisions [26]. Discrimination occurs when a disabled person is treated unfavourably because of their disability (here a rare disease) and this cannot be proved to represent a proportionate means of achieving a legitimate aim (section 15). Other features are summarised in Additional file 2: Table S2.

Disability legislation was recently used to overturn a recommendation by NICE. In *Eisai v NICE*, the High Court found discrimination against patients with Alzheimer’s disease after they were required to perform a mini mental state assessment to qualify for the drug Aricept, as this requirement had inadequate regard to those with atypical forms of the disease [27,28]. The claimants were able to demonstrate that certain patients were especially disabled by disease onset and that their cases were therefore unlike those of the majority of patients with this dementia. Similarly, patients affected by rare diseases are small groups suffering from particular genetic conditions, and there may be further subgroups within each disease of particularly strongly affected individuals.

The High Court has overturned another recent decision after withdrawal of support to patients with severe learning disabilities by a city council [29]. Although the Court did not wish to set priorities on behalf of the council, it concluded there had been a failure to consider the impact on disabled persons and no attempt made to make savings elsewhere [29]. By implication, funding for health-preserving orphan diseases cannot be curtailed without seeking to make savings elsewhere first. The Court has also affirmed the legal duty to promote equality of opportunity and to consider disabilities even where that involves treating the disabled more favourably [30], which augers in favour of allocating a larger per capita share of the health care budget to those affected by rare diseases.

### National and organisational constitutions

National constitutions may additionally prescribe rights to treatment. The French and German constitutions are thought to contain a legal obligation to assist individuals in danger, which could apply to the development of life saving treatments [31]. The UK does not have a written constitution but the NHS Constitution for England, underpinned by the Health Act 2009 and earlier legislation, is committed to non-discrimination and respect for human rights, and provides in particular six rights which can protect those with orphan disease. These are set out in Additional file 3: Table S3.

### Judicial review

Judicial review is a process for challenging the procedural basis on which a decision is made by a public authority [32,33]. Though traditionally aimed at ensuring the public body considers the necessary factors [34], the English Courts have broadened their scope in cases where human rights [33,35] or a “life or death decision” [36] are involved, as is the case with healthcare.

Indeed, the Courts have frequently overturned local authority decisions in favour of patients to ensure support or treatment is provided [29,30,33,37-40]. Patients’ requests for judicial review have also succeeded where the only available and clinically effective treatment has been denied [41], or when there have been insufficient clinical grounds for distinguishing between different members of a group that was in principle eligible for the drug, as was the case with the breast cancer treatment herceptin [36].

The courts have not found against allocating priorities per se, provided that (i) the policy genuinely recognises the possibility that there could be an overriding clinical need and requires each request for treatment to be considered on its individual merits, and (ii) the authority accurately assesses the nature and seriousness of each type of illness, determines the effectiveness of various forms of treatment for it, and gives proper effect to these assessments in the formulation and individual application of its policy [42]. Likewise, these are key elements of a sound funding policy for rare disease treatment and should feature in the revised funding guidelines for orphan medicinal products.

### Tort

Rare disease patients may argue that denial of treatment breaches tort law – a gap-filling area of the law which addresses any situations not captured by contract or criminal law. The English Court of Appeal has affirmed that in tort “it is the doctor’s duty to provide a treatment that he considers to be in the interests of the patient and that the patient is prepared to accept” [43]. As health care reform will see doctors taking a leading role in resource allocation, the duty to exercise reasonable skill and care [44,45] will likely apply to resource allocation. Health authorities are vicariously liable for any negligence on part of a doctor under tort law [45,46], and can also be directly liable for failure to take care in providing treatment [45,47] although, at least in the context of available medical equipment, the Court will pay some regard to budgetary constraints [45,48].

Patients affected by rare diseases could therefore seek redress for a doctor’s and/or hospital’s negligent denial or withdrawal of treatment. But litigation is strenuous and expensive. The preferred route would be for guidelines to be established as to when an orphan product should be reimbursed, to guide doctors in making a judgment as to whether to prescribe, whenever they judge it to be in the interest of a particular patient. Such decentralized assessment is helpful when the clinical situation is highly patient specific. In this regard, “interest” may be broader than proof of clinical efficacy: for some rare diseases such as Niemann-Pickdisease type C, where the approved therapy may stabilise a patient’s condition rather than improve it, but provision is imperative in order to sustain the patient until a more efficacious therapy is developed.

### Human rights legislation

Finally, a persuasive argument can be advanced that the European Convention on Human Rights (ECHR) demands the supply of orphan medicinal products to those in need. Central is Article 2 which provides that “everyone’s right to life shall be protected by law”. Other elements are summarised in Additional file 4: Table S4.

The European Court of Human Rights has specifically addressed orphan drug reimbursement in *Nitecki v Poland*. Mr Nitecki was diagnosed with motor neuron disease and prescribed Rilutek^TM^ with the Polish health authority agreeing to cover 70% of the drug cost [49]. However, the patient could not afford his 30% share and argued that refusal to refund fully violated his right to life under Article 2, as inability to take prescribed dose could result in untimely death.

However the Court found that the failure to cover the remaining 30% did not amount to a breach of Article 2, “bearing in mind the medical treatment and facilities provided to the applicant, including a refund of the greater part of the cost of the required drug” [49]. By implication, had the patient received *no* medical treatment or facilities, or less than a “greater part of the cost of the required drug”, Article 2 might have been violated. This point could assist patients faced with an authority unwilling to reimburse 50% of cost or denying access altogether. The courts would likely also consider the actual cost of any regimen: for example 30% of the drug treatment for Pompe could total £150,000 per annum [5] – something less than 1% of the UK population could afford [50], and would in effect amount to a denial of treatment, thus breaching Article 2. Indeed, the expectation that a patient cover a prohibitively large percentage of the cost arguably violates the NHS Constitution which provides care based on clinical need rather than ability to pay (see Additional file 3: Table S3).

The English Courts have affirmed that Article 2 would “plainly” be violated if a “National Health doctor were deliberately to bring about the death of a competent patient by withdrawing life-prolonging treatment contrary to the patient’s wishes” where the treatment will provide overall clinical benefit [43]. The case concerned the right to care at the end of life, and the courts would likely take an even stronger view for treatments which are life prolonging and at the start of life. Further, despite the pronouncements in *Osman v UK*[51], the text of Article 2 does not allow states to derogate from the right to life on fiscal grounds. Article 2 therefore appears to offer a promising platform for justifying access to treatments.

## Objections for funding orphan medicinal products

Despite the protections enshrined in law, the right of patients with rare diseases to access treatment has been questioned. Here we counter some potential objections to our analysis.

### Rarity not valued by society

It has, incredibly, been argued that there is inadequate evidence that society values equal opportunity for citizens affected by rare diseases to justify their high cost of treatment [52].

This is inaccurate, as shown by orphan drug enactments throughout the world, drawn up and ratified by parliaments representing a majority of each population, including the EU. The human rights and disability legislation considered above are further if indirect legal pronouncements of this view. These legal expressions of the society show rarity is to be valued.

Further, while pressure to reduce excessive cost must be exerted [14], current price levels result directly from the marketing monopoly provided by the orphan drug acts. It can be argued that the enactment of the EU Regulation 141/2000 without any price caps implies that society is willing to forego traditional cost-effectiveness measures for orphan medicinal products. One might respond that the financial impact of foregoing price caps was not known at the time. However, by the time the EU came to enact Regulation 141/2000, the US Orphan Drug Act had been in place for 17 years, and the problem of “unreasonably high” orphan drug costs had been aired even before the US House of Representatives [53]. Thus the impact of not capping prices was – or should have been – well known in Europe.

### Rare disease not a disability

It may be suggested that patients with rare diseases are not legally disabled as such legislation has traditionally been concerned with, for example, the use of wheelchairs in public transport vehicles – indeed the Equality Act devotes much attention this. However the Act defines disability to include progressive conditions, and no distinction is drawn between life threatening or chronically debilitating orphan diseases, and other progressive conditions such as multiple sclerosis identified by the Government as disabilities [24]. Indeed, rare diseases are understood to fall within the definition of disability under the United States Social Security Act [54,55].

### The cost of orphan drugs is unusually high for a disability

The public justifiably spends very substantial sums on providing wheelchair access. It would be unthinkable to propose limiting access to the British Museum to wheelchair users, or to apply a QALY-metric to see if such access is cost effective. Society is reluctant to cost certain things, even when lives are not at stake. No sound distinction can be drawn between a wheelchair-bound patient whose disability may never improve, and a patient with an orphan disease whose treatment only stabilises their clinical condition and does not reverse it (although many orphan drugs demonstrate such efficacy). Challenging funding for high cost orphan treatments therefore rests on an unjustifiable double-standard.

### Floodgates of disability

Innumerable conditions could be characterised as a disability, allowing access to rare disease and other treatments under the disability (and human rights) legislation irrespective of cost. This would open the flood gates of reimbursement for various conditions. The correct view must be that it is for each patient or group to argue why their condition should be classified as a disability (and patients need not have special public appeal or be well organised to so argue – most of the legal cases we showcase above have been brought by single individuals). Should they, too, succeed, this is a legitimate result of the wording of the legislation. If costs become untenable, this must be addressed on a governmental level, and not at the expense of an individual disabled citizen or small patient group.

### Disproportionate burden

Finally, some may contend that orphan treatments place a disproportionate burden on health services. This is a limitation felt acutely by the Courts when reviewing decisions of public authorities: “it is for the authority, not the court, to allocate resources within its limited budget to the best advantage of its many patients” [56,57]. The Court has suggested that had a trust refused Herceptin treatment on the basis of budget constraints rather than personal characteristics, “it would be very difficult, if not impossible, to say that such a policy was arbitrary or irrational” [36]. Similarly, in granting access to Avastin, the High Court indicated that it might have found differently if resource scarcity had been a “decisive feature” [41].

Our response is six-fold.

First, prices flowing from the EU Regulation 141/2000 are arguably not so disproportionate as to prevent reimbursement. After all, the legislation was approved without price caps. EU citizens therefore foresaw and accepted the impact of orphan medicinal products on the public purse (subject to the broader legal framework of the EU, including the need to avoid abusive pricing [14]).

Second, in assessing the budget impact of a rare disease therapy, the burden of the cost should not be considered only at the present date, but over the lifetime of a patient. It is hoped that the prices will decrease after the 10 year exclusivity periods expire and competing therapies enter the market. Further, a healthier patient can partake in employment, pay taxes and become a net economic contributor despite the high cost of treatment.

Third, the first principle of the current UK commissioning regime is to source services from providers best placed to cater to patients’ needs [26]. In the orphan field the producer of a treatment is best placed to satisfy such needs: due to the monopoly regime, the choice is normally between the only existing and therefore best (and best value) treatment, and no treatment at all. The commissioning regime thus supports the funding of orphan products.

Fourth, despite the English Courts’ timidity in interfering with priority setting, they do expect local authorities to prioritise serious illnesses [42] and consider statutory obligations including the human rights and disability provisions reviewed above. These dual considerations place orphan treatments at the top of any priority setting list.

Fifth, the Courts find it easier to overturn an authority’s denial of treatment where the impact on resources is less marked. This is unjustified. If society and its courts prioritise low-cost therapies, it allows morally arbitrary factors, such as disease prevalence, to determine whether a person can obtain full health.

Finally, every health authority decision carries an opportunity cost and impacts on resource allocation. Courts must therefore be courageous and not deny treatment or judicial review simply to avoid impacting on political resource allocation. Priority setting can be discriminatory and must comply with legal frameworks – a proper oversight role for the Courts. Were this to be otherwise, simply justifying decisions on budgetary grounds could place all decisions of a public authority outside the scope of human rights and discrimination laws, and the remit of judicial review. This is not the intention of the European or United Kingdom Parliament.

## Conclusion

Using the UK as an example, we have set out five legal approaches to safeguard the treatment of orphan diseases. While they together make a compelling legal case, judicial proceedings can be expensive, time-consuming and strenuous, especially while struggling with illness. This legal framework is therefore best used to negotiate national reimbursement guidelines for the European Union. Vulnerable rare disease patients on expensive treatments may become the first target of national spending cuts when ‘austerity measures’ are applied. It is therefore imperative that pricing and purchase decisions are made with full understanding of the legal protections available to these patients.

## Competing interests

All authors will complete the Unified Competing Interest form at http://www.icmje.org/coi_disclosure.pdf (available on request from the corresponding author) and declare no support from any organisation for the submitted work. The following financial relationships with organisations might have an interest in the submitted work in the previous three years: HIH and JCPR have received funding from the European Gaucher Alliance and Susan Lewis Memorial Fund, and HIH from the Helen Manuel Foundation. JCPR has attended symposia and received hospitality from Shire Human Genetic Therapies and speaking fees from the Genzyme Corporation and Actelion Pharmaceuticals. JM has received travel grants to attend industry sponsored meetings from Genzyme, Shire, Protalix and Pfizer. The EGA of which JM is Chairman receives unrestricted grants to support its activities from Genzyme, Shire, Protalix, Pfizer, Amicus and Actelion. The UK Gauchers Association has received sponsorship from Genzyme, Shire and Protalix for its UK activities and following his retirement as Chairman of the UK Gauchers Association, Genzyme presented an unrestricted and unsolicited donation to the UK Gauchers Association in his honour to be used to support Gaucher related activities at his discretion with agreement of the Board of the UK Gauchers Association. TMC has receives research support from the UK government, as well as unrestricted research grants from Genzyme and Shire, and he advises pharmaceutical companies engaged in the orphan disease setting, including Actelion, Genzyme, Shire, Amicus Therapeutics, and Protalix Biotherapeutics, receiving speakers’ fees and travel costs. HIH has had two younger sisters die of an orphan disease. There are no other relationships or activities that could appear to have influenced the submitted work.

## Authors’ contributions

JCPR conceived of the idea of applying disability legislation to the orphan funding problem. HIH conceived of the other ideas and developed the legal analysis. All authors have contributed to the legal, scientific and clinical analysis and their application to the orphan context. All four authors wrote the paper. TMC acts as guarantor for this work. All authors read and approved the final manuscript.

## Authors’ information

HIH is a lawyer with a background in philosophy, politics, and economics. JCPR is a medical doctor with a research interest in orphan diseases. JM is a lawyer, Chairman of the European Gaucher Alliance and Director and Co-Founder of UK Gauchers Association, Trustee of the Helen Manuel Foundation and was formerly a patient representative to the UK Department of Health National Commissioning Group on Lysosomal Storage Disorders. TMC is a physician who cares for patients with rare disorders. The views expressed here are the authors’ own and do not represent the view of any firm, company or charitable trust.

## Supplementary Material

Additional file 1: Table S1 UN Convention on the Rights of Persons with Disabilities [58]. Click here for file

Additional file 2: Table S2 United Kingdom Equality Act 2010. Click here for file

Additional file 3: Table S3 NHS Constitution [59-62]. Click here for file

Additional file 4: Table S4 European Convention of Human Rights [63,64]. Click here for file
