# Supplementary material for: The legal imperative for treating rare disorders
Source: Orphanet J Rare Dis. 2013 Sep 6;8:135. doi: 10.1186/1750-1172-8-135 (PMC4016581; doi:10.1186/1750-1172-8-135)
Supplement: Additional file 1: Table S1 — UN Convention on the Rights of Persons with Disabilities [58]. [file 1750-1172-8-135-S1.doc]

Additional file 1: Table S1 **UN Convention on the Rights of Persons with Disabilities [58].**

| **Description** | **Provision** | **Commentary** |
| --- | --- | --- |
| **Definition of disability** (Article 1 and Recital (e)) | “Disability” is not defined in the Convention but is recognised as an “evolving concept” which includes “long-term physical, mental, intellectual or sensory impairments” which may hinder a person’s full and effective participation in society on an equal basis with others. | Orphan diseases are life-threatening and chronically debilitating conditions [12] which result in long-term physical and often mental impairments which hinder patients’ participation in society on an equal basis with other EU citizens. |
| **Discrimination** (Articles 2 and Article 25(f)) | As noted above, discriminatory denial of health care or health services on the basis of disability is not permitted. “Discrimination on the basis of disability” is defined as  *any distinction*, *exclusion or restriction on the basis of disability which has the purpose or effect of impairing or nullifying the recognition*, *enjoyment or exercise*, *on an equal basis with others*, *of all human rights and fundamental freedoms in the political*, *economic*, *social*, *cultural*, *civil or any other field*. *It includes all forms of discrimination*, *including denial of reasonable accommodation* (on which see Article 5 below). | A compelling case can be made that it is discriminatory to deny medical treatment and to ignore the “more intensive support” (Recital (j)) required by patients affected by rare diseases. These conditions have been labelled “orphans” because of the history of neglect due to their rarity. As a result, patients may suffer from (i) delayed access to diagnosis [13], (ii) lack of medication, as presently only 1% of rare diseases benefit from commercially available treatment [58], (iii) comparatively lower effectiveness of treatments for some rare conditions such as Fabry disease, and (iv) effects of insufficient patient numbers and clinical evidence to allow a full assessment of the value of new medicines [5,6]. The EU has accordingly identified orphan diseases as a “priority” [12]. |
| **Compliant laws and availability of goods** (Article 4 and Recital (o)) | The Convention does not merely require states to *refrain* from discriminatory conduct, but imposes on them *positive obligations* to eliminate discrimination. They are required to “adopt all appropriate legislative, administrative and other measures for the implementation of the rights recognized” in the Convention and to “modify or abolish existing laws, regulations, customs and practices that constitute discrimination against persons with disabilities”. The States must also promote the research, development, availability and use of “goods”, “services” and “new technologies” (with priority on technologies at an “affordable cost”) to meet the specific needs of disabled persons. In performing these activities, the States must “closely consult with and actively involve persons with disabilities, including children with disabilities, through their representative organizations”. | In the UK, this translates *inter alia* to a need to design the new “value-based” health assessment metric so as to eliminate and avoid discrimination of those suffering from rare diseases, and to provide orphan medicinal products. If only one treatment exists due to the statutory monopoly position of a company obtained under Regulation 141/2000, prioritising a treatment over another on the basis of “affordability” may not be possible. The consultation requirement demands the involvement of patients in the drafting of reimbursement and pricing guidelines and reimbursement decisions for orphan products. |
| **Equality** (Articles 2 and 5) | Article 5 imposes a duty to promote equality, which includes taking “all appropriate steps” to provide “reasonable accommodation”. This means “necessary and appropriate modification and adjustments not imposing a disproportionate or undue burden” to ensure that those with disability can enjoy and exercise on an equal basis with others all human rights and fundamental freedoms. | The duty of reasonable accommodation arguably comprises both the duty of arriving at appropriately adjusted health economics or other metrics for rare diseases, as well as providing patients with orphan medicinal products. It might be argued that the current high prices impose a “disproportionate or undue burden” on the Member State national health care systems. We share the view that effective downward pressure must be exerted. However, in the meantime, the current price levels are the result of Regulation 141/2000 which binds Member States. Consequently, the *process* resulting in the current pricing is proportionate, even if the prices themselves are not. |
| **Habilitation and rehabilitation** (Article 26) | This demands effective and appropriate health services to enable persons with disabilities to attain and maintain maximum independence, full physical, mental, social and vocational ability, and full inclusion and participation in all aspects of life. These must begin at the earliest possible stage. | These appropriate and ambitious goals set a concrete measure of success for Member States’ efforts to ensure equal treatment of their disabled citizens. Some rare diseases, such as Gaucher Type I, can already be treated so as to enable patients to reach a full degree of independence and physical, mental and vocational ability if the treatment is started early enough. In the case of rare diseases for which less effective treatment is currently available, Article 26 translates to a right to the maximum level of independence and physical, mental, social and vocational ability that each patient is able to obtain with the benefit of available medication. |
