# Supplementary material for: The legal imperative for treating rare disorders
Source: Orphanet J Rare Dis. 2013 Sep 6;8:135. doi: 10.1186/1750-1172-8-135 (PMC4016581; doi:10.1186/1750-1172-8-135)
Supplement: Additional file 2: Table S2 — United Kingdom Equality Act 2010. [file 1750-1172-8-135-S2.doc]

**Additional file 2: Table S**2 United Kingdom Equality Act 2010

| **Part** | **Duty** | **Commentary** |
| --- | --- | --- |
| Part 1, Section 19: indirect discrimination | This is found where a provision, criterion or practice which is discriminatory, places persons with whom the disabled person shares the characteristic (rare disease) at a particular disadvantage when compared with those with whom they do not share it, and it cannot be shown to be a proportionate means of achieving a legitimate aim. The Alzheimer’s case above is an illustration of this. | An authority making funding decisions would have to show that the application *inter alia* of standard cost-effectiveness criterion to orphan medicines is proportionate. |
| Part 3: exercise of public function | Part 3 makes it unlawful to discriminate against a person when exercising a public function. A service-provider must not, in providing the service, discriminate against a person (a) as to the terms on which it provides the service to them; (b) by terminating the provision of the service to them; (c) by subjecting them to any other detriment (Section 29(2)).  Provision of a service includes the provision of (i) goods or facilities (Section 31(2)) and, expressly, (ii) “medical treatment on the NHS” [22]. The Act would therefore appear to prohibit discrimination among other things in the way in which priority setting decisions are made. More concretely, limb (b) appears to prohibit the withdrawal of treatment.  Part 3 also imposes an overarching duty to make “reasonable adjustments” *inter alia* where a provision, criterion or practice of the authority puts a disabled person at a substantial disadvantage (Sections 20(2 and 3) and 31(9); Schedule 2(2)). This would appear to include the duty to apply a cost effectiveness formula which does not put rare disease patients at such a disadvantage. | (1) As the Act prohibits public bodies from taking steps which they have no power to take (Schedule 2(8)), it is imperative that the Government’s value based pricing model be designed to provide the Government, NICE and health care providers with the power to authorise the provision of orphan medicinal products on appropriate grounds.  (2) The exemptions pertaining to health care concern blood services, health and safety and care within a family, and do not exclude the provision of treatment from the ambit of the Act (Section 31(10), Schedule 3(13), (14) and (15)).  (3) Patients could bring enforcement action (claim for breach of the Act and/or judicial review) under this section in county courts (Section 114). |
| Part 11: promotion of equality | Part 11 establishes a general duty on public authorities to have due regard, when carrying out their functions, to the need to eliminate unlawful discrimination and to advance equality of opportunity [22]. It is clear that rare disease patients are not able to pursue educational, career and other life opportunities on a par with the remainder of the population without effective treatment.  Section 149 includes a positive duty to take steps to meet the needs of persons who share a disability as compared with persons who do not (Section 149(3)(b)), which arguably involves the provision of treatment (see also Section 158). The English High Court has recently found a breach by a public body of the earlier equivalent of Section 149, in the context of a person detained with a view to deportation who did not receive adequate treatment for his psychiatric illness while detained [38]. | (1) Compliance with the duties in this section is permitted to involve treating some persons more favourably than others (Sections 13(3) and 149(6)), in this case allocating more resources to the funding of orphan medicinal products than is ordinarily allocated per capita in the health care system. Some argue that providing expensive treatments to those in need is discriminatory in favour of those who have expensive-to-treat conditions [8]. The import of the Act is that this argument has no legal basis.  (2) Individuals cannot enforce Part 11 in the courts, for example by seeking damages. Part 11 duties are, however, enforceable by way of judicial review (Section 156). |
