# Supplementary material for: The legal imperative for treating rare disorders
Source: Orphanet J Rare Dis. 2013 Sep 6;8:135. doi: 10.1186/1750-1172-8-135 (PMC4016581; doi:10.1186/1750-1172-8-135)
Supplement: Additional file 3: Table S3 — NHS Constitution [59-62]. [file 1750-1172-8-135-S3.doc]

Additional file 3: Table S3 NHS Constitution [59-62].

| **Right** | **Description** | **Guidance in the NHS Handbook and Appendix** |
| --- | --- | --- |
|  | Right to receive NHS services free of charge, apart from certain limited exceptions sanctioned by Parliament. This right has recently been summarised as resource allocation “based on clinical need rather than ability to pay” [26]. | Part 1 of the NHS Act 2006 sets out the primary duty on the Secretary of State to promote a comprehensive health service and to provide or secure the provision of services for that purpose free of charge, unless charges are expressly provided for. The Secretary of State can make for example regulations imposing prescription charges, but the authors are not aware of any having been enacted for rare diseases [59,60,61]. Patients’ requests to supplement their NHS cancer care with privately purchased pharmaceutical products have triggered national debate as to whether this would contradict the spirit of the NHS[62]. |
|  | Right to access NHS services, not to be refused on unreasonable grounds. | NHS services “will always be available for the people who need them”. Equality legislation provides that “unreasonable” and unlawful grounds of denial include those based on disability (pages 18, 119). Moreover, Primary Care Trusts’ and NHS trusts’ decisions must be reasonable and procedurally fair (page 119). |
|  | Right not to be unlawfully discriminated against in the provision of NHS services including on grounds of gender, race, religion or belief, sexual orientation, disability (including learning disability or mental illness) or age. | See above. Further, discrimination may be contrary to Article 14 of the ECHR given the right to life under Article 2 of the ECHR (page 121). |
|  | Right to drugs and treatments that have been recommended by NICE for use in the NHS, if deemed clinically appropriate by the patient’s doctor. | Primary Care Trusts have a legal obligation to comply with such Directions (section 8 of the NHS Act 2006), although this is set to change from 1 January 2014. |
|  | Right to expect local decisions on funding of other drugs and treatments to be made rationally following a proper consideration of the evidence. | Such decisions are required by administrative law to be rational and procedurally fair (page 125). Of key importance is to design a fair procedure for the assessment of orphan medicinal products as part of the Government’s value based pricing metric. |
