# Supplementary material for: The legal imperative for treating rare disorders
Source: Orphanet J Rare Dis. 2013 Sep 6;8:135. doi: 10.1186/1750-1172-8-135 (PMC4016581; doi:10.1186/1750-1172-8-135)
Supplement: Additional file 4: Table S4 — European Convention of Human Rights [63,64]. [file 1750-1172-8-135-S4.doc]

Additional file 4: Table S4 European Convention of Human Rights [63-64].

| **Description** | **Provision** | **Commentary** |
| --- | --- | --- |
| **Inhuman or degrading treatment** (Article 3) | Case law suggests that withdrawal of treatment can breach Article 3 in “exceptional” circumstances which are subject to “rigorous scrutiny” by the Courts [63].  The European Court has held in *D v United Kingdom* that deportation of a terminally ill AIDS sufferer from the United Kingdom to his native island of St Kitts would breach Article 3 because he was exposed to the risk of dying in very distressful conditions due to the lack of effective treatment [63]. It also found in the case of a mentally ill prisoner in *Keenan v United Kingdom* that the lack of “appropriate medical care may amount to treatment contrary to Article 3” [37], relied on recently by the English High Court to find a breach of Article 3 in similar circumstances [38,39].  While the English Court of Appeal has (prior to enactment of the Human Rights Act 1998) opined that Article 3 is not triggered in questions of a health authority’s allocation of finite funds [42], the judicial opinion appears to have evolved. The High Court has recently applied the test in the case of a terminally ill Portuguese citizen who had six months to live and was denied accommodation and support in the UK on the basis that he could seek these in Portugal [33]. The Court found that the exceptional circumstances were satisfied because the patient was at the end of his life, and would face an “undignified and distressing” end in Portugal, including delay and difficulty in obtaining accommodation and treatment for his HIV and cancer [33]. | Given the focus on patients at the end of their lives, it is unclear whether the Courts would consider Article 3 triggered where a patient seeking access to an orphan medicinal product is years rather than months away from death.  A parallel between the cases is that, like Mr D and the Portuguese Mr De Almeida, patients affected by rare diseases commonly have no other country in (or financial fund through) which to seek treatment.  Moreover, the text of Article 3 does not impose a criterion of imminent death. Indeed, the denial of life-saving treatment is potentially even more distressing, and therefore more inhuman or degrading, where the patient faces living with an untreated and debilitating condition for years rather than months.  The European Court has in fact stressed the need to retain “flexibility” in addressing the contexts to which Article 3 may apply [63], and the English High Court itself has emphasised that the focus is on the *effect* of the decision on the patient [33]. Article 3 may therefore aid those seeking access to orphan treatments. |
| **Right to private and family life**  (Article 8) | Article 8 secures respect for a person’s private and family life. This may involve the provision of medical treatment [33,64]. The European Court has however been wary to establish a positive obligation on the state to provide benefits because questions of budget allocation fall within the political domain [64].  Further, Article 8(2) permits “interference by a public authority” if the interference “is in accordance with the law and is necessary in a democratic society in the interests of … the economic well-being of the country … for the protection of health … or for the protection of the rights and freedoms of others”. This involves striking a proportionate balance between the rights of the individual and the interests of the community [33], in which exercise states have broad discretion [64].  Given these reservations, it is thought that Article 8 imposes no positive obligation to provide medical treatment [28], and the High Court recently declined to find Article 8 breached by a Primary Care Trust which had denied bariatric surgery to a patient [64].  However, the English High Court held recently in the case of the Portuguese cancer patient (see Article 3 above) that denial of accommodation and support also breached Article 8. It relied particularly on three factors. First, the financial burden on the public purse was limited as the patient had a limited life expectancy. Second, the patient had previously worked in the UK and not relied on public resources for his welfare. Third, the cost that would have been incurred by the Borough in repatriating the patient to Portugal was so large as to result only in a minimal saving, as compared with allowing the claimant to stay in the UK [33,39]. The Borough therefore failed to show that deportation was necessary in a democratic society. | Applying the High Court’s reasoning in *De Almeida* to the orphan context:  (1) The cost assessment of orphan drugs must take into account the cost of the alternative, in other words treating disease manifestations rather than providing the orphan medicinal product. For example in the case of a Gaucher patient this would involve costly blood transfusions, splenectomy, analgesia, hospitalisation and joint replacement surgery. The analysis should also consider the cost of the orphan drug over the patient’s life time, as the cost may decrease after the statutory monopoly of the producer expires and competing treatments enter the market.  (2) Where effective treatment exists, this also allows patients to participate fully in employment and thereby not rely on public benefits (although this should not be a qualifying criterion as some treatments are not effective enough to permit a patient to engage in gainful employment).  (3) The High Court’s consideration that a patient at the end of his life imposed a limited budget impact, is suspect: from the patient’s perspective it is morally arbitrary as to whether they are at the start or end of their lives.  We note for completeness that the Article 8 case law may need clarification in two respects:  (1) While the Court is correct in identifying that budget allocations are made within the political domain, the distribution of medical budgets must also comply with the European and domestic legal framework, including human rights and disability legislation considered below. The Courts can therefore not shy away from taking a position as to applicability of Article 8 in the context of health care resources.  (2) The qualification in Article 8(2) should not be based on utilitarian considerations of what maximises benefit for society as a whole, as this may sacrifice the rights of an individual and utilitarianism has been discredited as a sole basis of priority setting decisions in the medical arena. |
| **No discrimination**  (Article 14) | The European Court in *Nitecki* indicated that Article 14 could in principle be violated by a refusal to provide health care. However, it found that the right was not violated because the refusal to provide complete reimbursement was reasonable in the “present health care system which makes difficult choices as to the extent of public subsidy to ensure a fair distribution of scarce financial resources” [49]. | It is arguable that, in analysing Article 14, the Court in *Nitecki* overlooked the EU-assigned priority status of orphan conditions considered further in relation to disability legislation below. |
| **Application in the UK** | The ECHR has been implemented in the United Kingdom by the Human Rights Act 1998. Domestic legislation, such as the Equality Act considered below, must be read and given effect to in a way which is compatible with an individual’s rights under the Convention so far as it is possible to do so (Section 3(1)). It is also unlawful for a public authority such as the NHS to act in a way which is incompatible with a Convention right (Section 6(1)). An individual can bring court or tribunal proceedings against a public authority in contravention of the Act (Section 7), although in our view the more effective route to treatment is a dialogue between all affected parties, founded on the provisions of *inter alia* the ECHR and the disability legislation, to which we now turn. | - |
